# Royal Medical Benevolent College

**Published:** 1893-02-04

**Authors:** C. Holman, T. N. Hart Smith

**Affiliations:** Treasurer; Head Master


### Royal Medical Benevolent College.

The Royal College of Physicians, London, having agreed "That Instruction in Chemistry, Physics, Practical Chemistry, and Elementary Biology at Epsom College be recognised as fulfilling the requirements of the new regulations for admission to Parts I. and II. of the First Examination," we have received the following letter :—

It gives us great pleasure to send you a copy of the recommendation from the Board of Management of the conjoint Board, which has been approved and adopted by both the College of Physicians and College of Surgeons. This recognition of Epsom College must be a great boon to pupils passing through the school prior to entering on their curriculum at the hospitals.

All the elementary book work will now be got over during school life, at a time when lads need most aid in grasping the subjects they are learning, and during a time when the continuance of school discipline and the responsibilities falling to the seniors are so invaluable in the formation and consolidation of character. We hope to be able to send our students to their freer hospital life better prepared to meet the inevitable temptations they must be exposed to.

The advantages thus afforded will further benefit those boys who most require help. No important changes will be required in the course of study carried on at Epsom College. Abler boys who can look forward to taking a medical degree at London University or at Oxford or Cambridge have for years past been successfully trained in the requisite subjects for the Preliminary Scientific Examination. But those who, for various reasons, have sought to qualify in other ways for a medical career, have hitherto been obliged to leave us before they could receive the full benefits of our curriculum, and at far too early an age and with too immature powers to receive hospital instruction with profit. And that which will, we hope, enlist the sympathy of every member of the profession, especially that of the teachers in our great medical schools, is that such boys will now during the whole time of University or hospital life be free to learn those great subjects and see that practice which is in the future to fit them successfully to follow the practice of a profession to which they propose to devote their lives, and in which a healthy ambition will make them seek to rise to distinction.

Signed, C. Holman, Treasurer.
T. N. Hart Smith, Head Master.

